# Retraction Note: Anti-inflammatory Effects of Novel P2X4 Receptor Antagonists, NC-2600 and NP-1815-PX, in a Murine Model of Colitis

**DOI:** 10.1007/s10753-024-02143-x

**Published:** 2024-09-14

**Authors:** Vanessa D’Antongiovanni, Carolina Pellegrini, Laura Benvenuti, Matteo Fornai, Clelia Di Salvo, Gianfranco Natale, Larisa Ryskalin, Lorenzo Bertani, Elena Lucarini, Lorenzo Di Cesare Mannelli, Carla Ghelardini, Zoltan H. Nemeth, György Haskó, Luca Antonioli

**Affiliations:** 1 Department of Clinical and Experimental Medicine, University of Pisa, Pisa, Italy; 2 Unit of Pharmacology and Pharmacovigilance, Department of Clinical and Experimental Medicine, University of Pisa, Via Roma 55, 56126 Pisa, Italy; 3 Department of Translational Research and New Technologies in Medicine and Surgery, University of Pisa, Pisa, Italy; 4 Department of Neuroscience, Psychology, Drug Research and Child Health, Neurofarba, Pharmacology and Toxicology Section, University of Florence, Florence, Italy; 5 Department of Surgery, Morristown Medical Center, Morristown, NJ 07960 USA; 6 Department of Anesthesiology, Columbia University Irving Medical Center, New York, NY 10032 USA

**Retraction Note: Inflammation (2022) 45:1829-1847**

10.1007/s10753-022-01663-8

The Editor-in-Chief has retracted this article. After publication, concerns were raised regarding highly similar western blot lanes in Fig. 6e (lanes 3 and 4; 5 and 8). Additionally, in Fig. 2a, two groups of rats lost >30% of their body weight, and Figs. 2c and 3 show macroscopic and microscopic injury scores, but no representative images are included.

The Editor-in-Chief therefore no longer has confidence in the presented data and the ethics of this study.

The authors have not responded to any correspondence from the editor or publisher about this retraction.

